# Emergence of phenotypic plasticity through epigenetic mechanisms

**DOI:** 10.1093/evlett/qrae012

**Published:** 2024-03-27

**Authors:** Daniel Romero-Mujalli, Laura I R Fuchs, Martin Haase, Jan-Peter Hildebrandt, Franz J Weissing, Tomás A Revilla

**Affiliations:** Zoological Institute and Museum, University of Greifswald, Greifswald, Germany; Institute for Botany and Landscape Ecology, University of Greifswald, Greifswald, Germany; Zoological Institute and Museum, University of Greifswald, Greifswald, Germany; Zoological Institute and Museum, University of Greifswald, Greifswald, Germany; Zoological Institute and Museum, University of Greifswald, Greifswald, Germany; Groningen Institute for Evolutionary Life Sciences, University of Groningen, Groningen, The Netherlands; Department of Mathematics, Faculty of Science, University of South Bohemia, České Budějovice, Czech Republic; Czech Academy of Sciences, Biology Centre, Institute of Entomology, České Budějovice, Czech Republic

**Keywords:** phenotypic plasticity, epigenetics, methylation, evolution, limits, costs, cryptic variation, model, mechanism

## Abstract

Plasticity is found in all domains of life and is particularly relevant when populations experience variable environmental conditions. Traditionally, evolutionary models of plasticity are non-mechanistic: they typically view reactions norms as the target of selection, without considering the underlying genetics explicitly. Consequently, there have been difficulties in understanding the emergence of plasticity, and in explaining its limits and costs. In this paper, we offer a novel mechanistic approximation for the emergence and evolution of plasticity. We simulate random “epigenetic mutations” in the genotype–phenotype mapping, of the kind enabled by DNA-methylations/demethylations. The frequency of epigenetic mutations at loci affecting the phenotype is sensitive to organism stress (trait–environment mismatch), but is also genetically determined and evolvable. Thus, the “random motion” of epigenetic markers enables developmental learning-like behaviors that can improve adaptation within the limits imposed by the genotypes. However, with random motion being “goal-less,” this mechanism is also vulnerable to developmental noise leading to maladaptation. Our individual-based simulations show that epigenetic mutations can hide alleles that are temporarily unfavorable, thus enabling cryptic genetic variation. These alleles can be advantageous at later times, under regimes of environmental change, in spite of the accumulation of genetic loads. Simulations also demonstrate that plasticity is favored by natural selection in constant environments, but more under periodic environmental change. Plasticity also evolves under directional environmental change as long as the pace of change is not too fast and costs are low.

## Introduction

Phenotypic plasticity is a phenomenon found in all kingdoms of life. It is defined as the ability of an organism to adjust its phenotype in response to the local environment and its changes, without any change in the genome (Pigliucci, 2005). Theoretical models suggest that phenotypic plasticity evolves under variable environmental conditions, whenever environmental cues are reliable and, therefore, environments predictable and costs relatively low (Berrigan & Scheiner, 2004; Botero et al., 2015; Chevin et al., 2010; Lande, 2009, 2014; Pfennig, 2021; Scheiner & Holt, 2012; Scheiner et al., 2020). Controversially, when costs are negligible, plasticity—and behavior—can relax selection on genotypes and slow down evolutionary adaptation by genetic changes to permanently altered environmental conditions (Huey et al., 2003; Thibert-Plante & Hendry, 2011). Understanding fitness costs and limits of phenotypic plasticity requires considering the regulatory processes underlying phenotypic response patterns. Though some models cited above involve genetic-explicit individual-based simulations, the treatment of plasticity remains phenomenological, with no clear link to the action of the underlying plasticity mechanism. In nature, plasticity is typically mediated by regulatory processes (e.g., developmental switches, gene regulation, and neural or physiological control mechanisms, Sommer, 2020). A change in plasticity, therefore, reflects a change in these regulatory mechanisms.

This change in perspective (from the phenomenological to the mechanistic approach to plasticity) has major implications (van Gestel & Weissing, 2016; Wagner, 2005; Watson & Szathmáry, 2016). The mechanistic approach has led to (i) many-to-one genotype–phenotype maps and, therefore, mutational robustness of the wild-type phenotype (van Gestel & Weissing, 2016), due to different genotypes potentially displaying similar phenotypic responses, and (ii) enhanced evolvability (i.e., ability to respond to selection, Houle, 1992) by the uncovering of cryptic genetic variation when organisms are exposed to atypical environmental conditions. Cryptic genetic variation refers to genetic variation that has little or no effect on phenotypic variation under “normal conditions,” but that under atypical (stressful) conditions generates phenotypic variation (Paaby & Rockman, 2014). However, it is unclear whether cryptic genetic variation results from hidden developmental programs that evolved from past selection or subject to drift when silent, or whether it is a manifestation of limitations of the plasticity mechanism itself, or a combination of both (Laland et al., 2015; Parsons et al., 2020; Romero-Mujalli et al., 2021).

In this paper, we propose a novel mechanism for phenotypic plasticity based on epigenetics, of the kind that may be attained via DNA-methylation/demethylation (Duncan et al., 2022). In nature, patterns of DNA methylation have been associated with both environmental factors and phenotypic variation in populations experiencing different—and stressful—environmental conditions, and observed for a wide range of taxa (e.g., bacteria, Casadesús, 2016; yeast, Hattman et al., 1978; snails, Fallet et al., 2020; Smithson et al., 2020; Thorson et al., 2017, 2019; plants, Niederhuth et al., 2016; mammals, including humans, Li & Zhang, 2014). Furthermore, the role of methylation on gene expression (silencing/activation) has been extensively documented and is phylogenetically widespread (Adrian-Kalchhauser et al., 2020; Curradi et al., 2002; Greenberg & Bourc’his, 2019; Kribelbauer et al., 2020). Epigenetic markers (e.g., methylation of the C5 positions in cytosines) can be induced by the environment and have been proposed as a candidate molecular mechanism underlying plastic phenotypic responses (Angers et al., 2020; Massicotte & Angers, 2012). However, the evolution and action of such a mechanism remains unknown since most of the empirical support is correlative (Richards et al., 2017; Schlichting & Wund, 2014).

In our model, the behavior (bind/release) of the epigenetic markers (methyl groups) follows random motion, but the frequency of events (methylation/de-methylation) is sensitive to organism stress (trait–environment mismatch). The epigenetic modifications (hereafter, epigenetic mutations or simply, epimutations) affect fitness-affecting polygenic traits of individuals. This is a novel approach that connects stimulus (stress) and response (trait expression) through feedback loops. On the one hand, this mechanism imitates developmental learning by trial and error, but on the other hand, it is also vulnerable to developmental noise leading to maladaptation. Using individual-based simulations, we study the role of epigenetic modifications on the performance of individuals and populations, as well as the evolution of the optimum level of plasticity under scenarios of periodic or directional environmental change.

## Model and methods

We implemented an individual-based model where N individuals i=1,…,N have phenotypes determined by a single *response trait*$x_{i}\left(t\right)$ that must match an environmental parameter p(t) across multiple generations t=1,2,3,…. Natural selection favors the reproduction of phenotypes that are best at matching the environment. Response traits are genetically determined, but can be altered during a finite development period (there are two-time scales: *developmental* nested in *generational*). Such phenotypic plasticity (also called developmental plasticity; Botero et al., 2015) is enabled by epigenetic modifications, with adaptive rates $\mu_{e}$ controlled by a *sensitivity trait*ω which also can be genetically coded, and thus evolve across generations.

**Figure 1:**
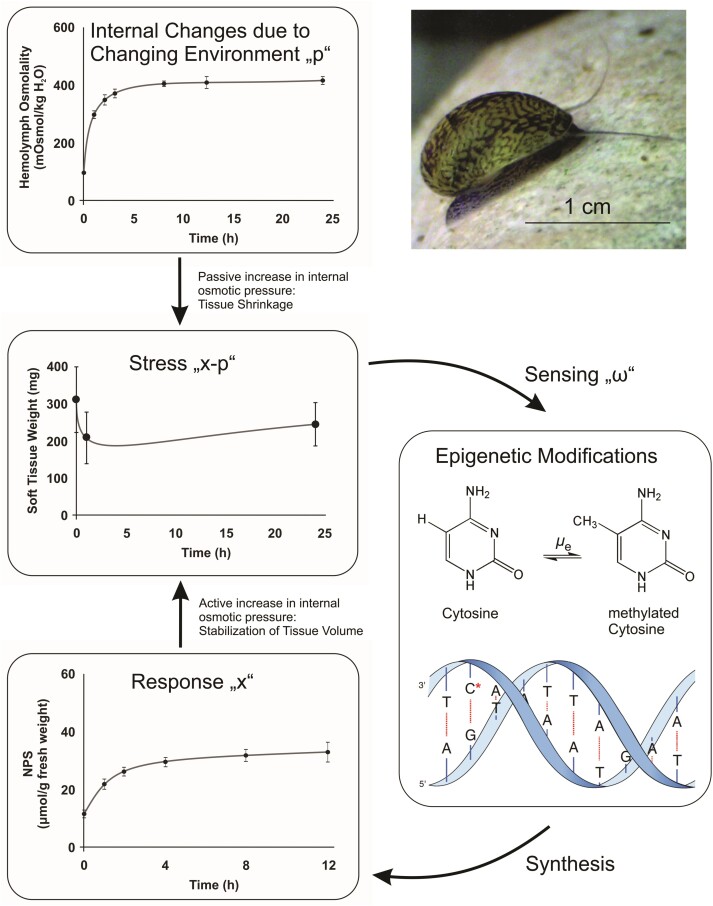
Conceptualization of our model, taking phenotypic plasticity in the snail *T. fluviatilis* as an example. An increase in the osmolality “p” of the environment (e.g., hypersalinity) leads to an osmotic imbalance associated with dehydration (tissue shrinkage). Stress (“x−p”) sensing (“ω”) modulate rates (“$\mu_{e}$”) of epigenetic modification via DNA methylation (e.g., cytosine-methylation, C* in the DNA structure)/demethylation of multiple loci encoding proteins involved in the synthesis of metabolites (e.g., ninhydrin-positive substances, NPS). Increasing amounts of NPS represent the organism’s response “x” which adjusts the internal osmotic situation of the animal to the external conditions, thereby stabilizing tissue volume. The organism’s reaction alters its phenotype, but not its genotype. The photograph of a live freshwater snail has been taken by Amanda Wiesenthal. The originals of the redrawn graphs appeared in Symanowski and Hildebrandt (2010). The image of the DNA structure has been adapted from Hildebrandt et al. (2021).

Figure 1 illustrates the plasticity mechanism by the stress response of *Theodoxus fluviatilis* (Neritidae), a dioecious euryhaline snail that occurs in freshwater lakes and streams in central Europe. For *T. fluviatilis*, as for other snails under osmotic stress, p may represent the osmolality of the environment, and x the internal osmolality achieved by the accumulation of free amino acids and urea. Thus, x=p corresponds to a favorable isotonic state; x<p corresponds to a hypotonic condition (internal milieu less concentrated than external medium) leading to dehydration; and x>p to a hypertonic condition (internal milieu more concentrated than external medium) leading to swelling.

Our model implements these modifications by simulating DNA-methylation/demethylation that activate/deactivate multiple loci randomly, with alleles that determine response traits additively. This mechanism could also work for a single response trait locus, with an activation degree (0%–100%) given by a variable number of methylated CpG sites. Both interpretations are conceptually and mathematically identical, we keep the loci view because it is in line with quantitative-genetics theory and models (Barton et al., 2017; Bürger & Lynch, 1995; Scheiner et al., 2020). Once matured, individual traits determine mating success, and parents are replaced by offspring. Alleles for $x_{i}\left(t\right)$ and $\omega_{i}\left(t\right)$ pass to the next generation (t+1) along with random mutations, but epigenetic modifications are lost, that is, no epigenetic inheritance (Jablonka, 2017). Natural selection acting directly on $x_{i}\left(t\right)$ and indirectly on $\omega_{i}\left(t\right)$ values takes place in environments with periodic or directional trends p(t) across generations.

### Response and sensitivity traits

This section deals with independent development of single individuals in a generation. Thus, individual and generational markers (i,t) are omitted in favor of notation economy. An individual’s response trait x is encoded by L diallelic loci as follows


$$x=\sum_{j=1}^{B}\left(l_{j1}+l_{j2}\right)+\sum_{j=B+1}^{L}\left(l_{j1}+l_{j2}\right)a_{j},$$ (1)

with allelic values $l_{jk}$ ranging from −∞ to ∞, that is, there are potentially infinite alleles per locus. k=1,2 denotes alleles from different parents. There are B non-plastic loci encoding a baseline trait value, and L−B plastic loci contributing or not, depending on activation bits or tags $a_{j}\in \left\{1,0\right\}$. The number of non-plastic B loci must be at least equal to 1; otherwise, if B=0, that is, all L loci are plastic, a scenario in which all loci become inactive would invoke an indeterminate trait value. This genotype-to-phenotype mapping implies that multiple genotypes can produce a common phenotype, but also a specific genotype can produce multiple phenotypes. To illustrate the first case suppose that $a_{j}=1$, then an individual can have a phenotype x=2 if: (a) both alleles equal to one at one locus, all others zero; (b) one allele equal to one at two different loci, all others zero; (c) all alleles equal to 1/L; (d) large variation in allele values (some positive and some negative), with an average value of 1/L. To illustrate the second case, consider L=10, B=1, and $l_{jk}=1$: the phenotype x could be any even number from 2 to 20 depending on the activation states of L−B=9 loci.

Plastic loci j=B+1,…,L experience epigenetic mutations over a development period of τ days. These consist of random deactivations $\left(a_{j}=1\to 0\right)$ and reactivations $\left(a_{j}=0\to 1\right)$. The probability of epigenetic mutation per (plastic) locus per day


$$\mu_{e}\left(x|\omega ,p\right)=1-\exp\left(-\omega {\left(x-p\right)}^{2}\right),$$ (2)

increases with the mismatch between the response trait and the environmental parameter p (Foster, 2007). The sensitivity of the response is modulated by a second trait, ω. Thus, the response can be plastic: the phenotype of individuals with ω>0 can be modified during development by random loci activations and deactivations.

The sensitivity trait ω is a critical parameter of (2). If it is neither too small nor too large, the frequency of epigenetic mutations is expected to decrease rapidly with |x−p|, making phenotype optimization by trial and error efficient. On the other hand, if ω is too large, epigenetic mutations can be too frequent even for small |x−p|, resulting in developmental noise and maladaptation. Thus, optimum ω values, that is, optimum levels of plasticity, are expected to result from natural selection. To account for this, we also consider ω a heritable trait, encoded by a single locus as follows


$$\omega =\frac{\left|\lambda_{1}+\lambda_{2}\right|}{s},$$ (3)

with allelic values $\lambda_{k}$ also in the −∞ to ∞ range. A scaling parameter s>0 is used to calibrate the simulations.

### Population dynamics and evolution

Evolution of response x and sensitivity ω traits occurs by natural selection acting on reproductive fitness, in populations with discrete, non-overlapping generations. A population of N individuals (i=1,…,N) is split into M=1/2N “males” (rounded to lowest integer) and F=N−M “females.” Each female mates once; its mate is drawn at random from the population of males (with replacement, implying that males can mate more than once). The number of offspring produced by the pair is drawn from a Poisson distribution with parameter $W_{\text{♂}}+W_{\text{♀}}$ (♂=1,…,M and ♀=1,…,F), that is, the sum of female and male fitness. Hence, the “fitness” of an individual corresponds to the individual’s effect on its expected number of offspring; this effect may include both survival and fecundity. We assume that male and female fitness is given by


$$W\left(x_{i},\omega_{i},N\right)=\left(1-\rho \left(x_{i}\right)\right)\cdot R\,\exp\left(1-\frac{N}{K}\right)-\omega_{i}C,$$ (4)

The term R⁢exp⁡(1−N/K) may be viewed as a baseline fitness, which is density dependent: R corresponds to the “intrinsic reproduction factor,” and the “carrying capacity” K is the population size above which baseline fitness drops below R. The $\left(1-\rho \left(x_{i}\right)\right)$ factor in front of R introduces fitness depression experienced due to stress, which is defined as


$$\rho \left(x_{i}\right)=1-\exp\left(-\frac{1}{2}{\left(\frac{x_{i}-p}{\gamma }\right)}^{2}\right),$$ (5)

where $x_{i}$ is the response trait value (1) at the end of the development phase and p is the environmental parameter value in the current generation. Stress is 0 if $x_{i}=p$, increases monotonically with the $\left|x_{i}-p\right|$ difference, and is bounded above by 1 (asymptote). The inflection parameter γ determines stress tolerance: small (large) γ corresponds to low (high) tolerance, that is, strong (weak) natural selection. Plastic individuals bear a fitness cost C per unit of sensitivity trait ($\omega_{i}$).

Each offspring inherits one allele from each parent at each locus. Allele values of the inherited haplotype are randomly selected from the corresponding parental locus (no linkage). Allelic values ($l_{jk},\lambda_{k}$, “Response and sensitivity traits” section) mutate with rate $\mu_{m}$ per locus per generation. Mutational effects $\left(\Delta l_{jk},\Delta \lambda_{k}\right)$ are normally sampled with zero mean and standard deviation $\sigma_{m}$. The offspring entirely replaces the parental population in the next generation. Keep in mind that since the offspring is the sum of a finite number (the number of females) of Poisson variables, extinction (the sum is zero) is possible.

Fitness is density-dependent in view of Equation (4), as well as frequency-dependent, because the fitness of couples (matings) combines heterogeneous male and female fitnesses.

### Environmental change

We assume that individuals experience a constant environment p through their lives, but changes occur between discrete generations t=1,2,… We consider two trends: *periodic* or *directional* (Botero et al., 2015; Bürger & Krall, 2004). Cycles of period T and amplitude A are modeled by sinusoidal functions


$$p\left(t\right)=p_{0}+A\,\sin\left(\frac{2\pi t}{T}\right),$$ (6)

and directional changes are modeled by the linear function


$$p\left(t\right)=p_{0}+\eta t,$$ (7)

where η is the rate of environmental change. The initial state $p_{0}$ is zero’ed at the start of the simulations. Both trends are deterministic (i.e., no form of environmental stochasticity is considered). Although previous studies have shown that the color of the stochastic noise affects the importance of phenotypic plasticity for populations under environmental change (e.g., Ashander et al., 2016), its effect on phenotypic plasticity is beyond the scope of this study.

### Simulations

We carried out the following simulation protocols for sexual diploid populations

1. **Effect of plasticity on development.** Follows the development of a population in a single generation. Individuals have distinct response traits $x_{i}$ updated according to (*Equation (1)*), but the sensitivity parameter ω is the same for all (with ω=0 as control case). This allows us to study the action and limitations of the plasticity mechanism.
2. **Effect of plasticity on populations.** Follows the evolution of response traits under allelic mutation and recombination. Like the first protocol, all individuals have the same fixed ω. This allows us to study how epigenetic-driven plasticity steers evolution under periodical or directional regimes of environmental change. Since individuals share the same ω, plasticity costs are meaningless here, so we set them C=0.
3. **Evolution of plasticity.** Like the second protocol, alleles for the sensitivity trait also mutate, and ω gets updated each generation according to (*Equation (3)*). Simulations start with ω=0 for all individuals, and we follow its evolution under different plasticity costs (C≥0). We consider constant environment scenarios, as well as periodical or directional changes.

Response trait alleles $l_{jk}$ were initialized by normal sampling with zero mean and variance $\sigma_{G}^{2}/2L$, where $\sigma_{G}=1$ is the standard deviation of traits in the population before development (first protocol) and evolution (second and third protocols). Sensitivity alleles were initialized $\lambda_{k}=0$ for the third protocol. The third protocol also considers a neutral marker (locus) with alleles initialized $\nu_{k}=0$, allowing us to distinguish the effects of natural selection from genetic drift. The allelic mutation rate ($\mu_{m}$) and effect ($\sigma_{m}$) are the same for all allele types. A list of the model’s variables parameters is shown in Table 1.

**Table 1:** Model variables and parameters.

Symbol	Description	Values
x	Response trait	Equation (1)
ω	Sensitivity trait	Equation (3)
p	Environmental parameter	Equation (6) or (7)
L	Number of response trait loci	10
B	Number of non-plastic loci	1
$l_{jk}$	Allelic value of response locus j from parent k	(−∞,∞)
$\lambda_{k}$	Allelic value of sensitivity locus from parent k	(−∞,∞)
$\nu_{k}$	Allelic value of neutral locus from parent k	(−∞,∞)
s	Sensitivity scale	42
τ	Development time	33
$\mu_{e}$	Rate of epigenetic mutation	Equation (2)
$\mu_{m}$	Rate of allelic mutation	$10^{-4}$
$\sigma_{m}$	Standard deviation of allelic mutational effects	1
$\sigma_{G}$	Standard deviation of traits before development	1
W	Individual fitness	Equation (4)
ρ	Individual stress	Equation (5)
γ	Stress tolerance	2.2
N	Population size	Non-negative
R	Intrinsic reproduction factor	1
K	Carrying capacity	1,000
C	Plasticity cost	0 to 1 in steps of size 0.1
T	Period of environmental cycling	200
A	Amplitude of environmental cycling	1, 4
η	Rate of directional environmental change	0.001, 0.004

## Results

### Effect of plasticity on development

**Figure 2:**
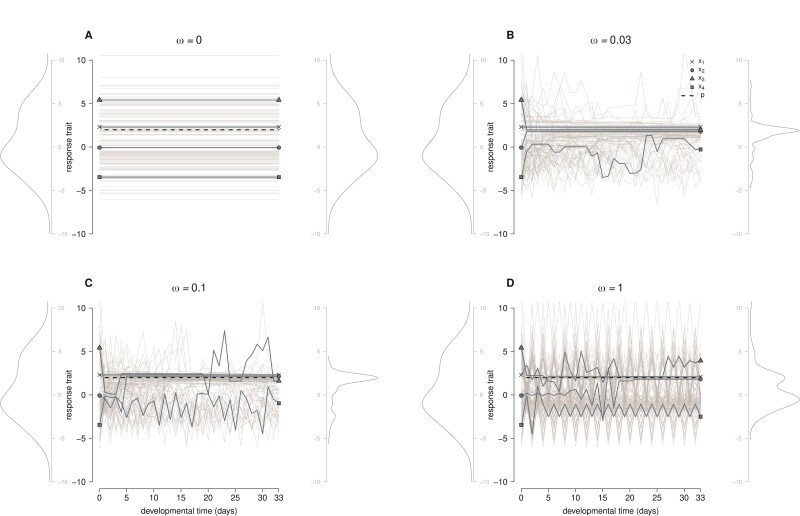
Development of response traits x (Equation (1)) for 100 individuals (the same in all panels). The environmental parameter is p=2, and the sensitivity trait is ω=0,0.03,0.1,1, from panels A–D. The development of four individuals (“cross,” “circle,” “triangle,” and “square”) is highlighted. Trait distributions at the start and end of development are displayed on the left and right sides of each panel, respectively. Parameters from Table 1.

We used single-generation simulations to demonstrate how epigenetic mutations enable a form of developmental learning that, by trial and error, adjusts phenotypes to current environmental conditions. Allelic values ($l_{jk}$) were normally sampled with mean zero and unit variance. Thus, response traits ($x_{i}$) are distributed approximately normally with a mean of zero at the start of development (notice probability density functions (p.d.f.) on the left sides of panels of Figure 2). All individuals share a common sensitivity trait value (ω). Plastic populations (ω>0) have a fair chance of matching phenotypes with the environment at the end of development (x mode ≈p), as long as sensitivity traits are moderate. This is shown in Figure 2B and C as reduction of phenotype variance (right p.d.f. narrower than left p.d.f.), compared with non-plastic populations in panel A (ω=0, flat lines). However, if the sensitivity trait is too large like in panel D, plastic phenotypes are less likely to match the environment (final p.d.f. as wide as starting p.d.f.).

Given the randomness of epigenetic mutations, phenotype plasticity does not progress in the same fashion for all individuals. Those starting with phenotypes close to the environmental parameter are unlikely to change at all during development, whereas those starting far from it display erratic trends toward the optimum phenotype value $x_{i}=p$, or may miss the target by a large margin. Interestingly, if the sensitivity trait is extremely high, many individuals display phenotype oscillations and cannot approach the optimum. This is because epigenetic mutations rates (Equation (2)) can get very close to one, causing alternating activation/de-activation at plastic loci. These behaviors are illustrated in Figure 2, where we singled out the same four individuals (“cross”, “circle,” “triangle,” and “square”) in all panels.

To summarize, the effect of epigenetic mutations according to our model is not uniform. Optimal phenotypes are more likely to develop for intermediate values of the sensitivity parameter. If ω or $\left|x_{i}-p\right|$ are too small, little to no activation changes take place; but if ω or $\left|x_{i}-p\right|$ are too large, the high frequency of epigenetic mutations makes phenotype–environment matching unlikely. In other words, loss of function by developmental noise is pleiotropic with plasticity (and this limits the evolution of plasticity, see “Evolution of plasticity” section). Intermediate ω values balance these tendencies: they make epigenetic mutations frequent enough at the start of development, which is useful to explore the phenotype space, and less frequent toward the end of development, preventing loss of optimality (Supplementary Figure A.1 shows a gradual decline of $\mu_{e}$ during development). We call this “learning-like behavior.”

Supplementary results show similar developmental trends if initial trait distributions are not normal, but asymmetric or bimodal (Supplementary Figures A.2 and A.3, respectively). These kinds of distributions could arise as a result of mutation and selection in past generations. We found that increasing the number of plastic loci (i.e., decreasing B, Supplementary Figure A.4) and the development time (increasing τ, Supplementary Figure A.5), raise the chances that optimal responses are found by developmental learning.

### Effect of plasticity on populations

**Figure 3:**
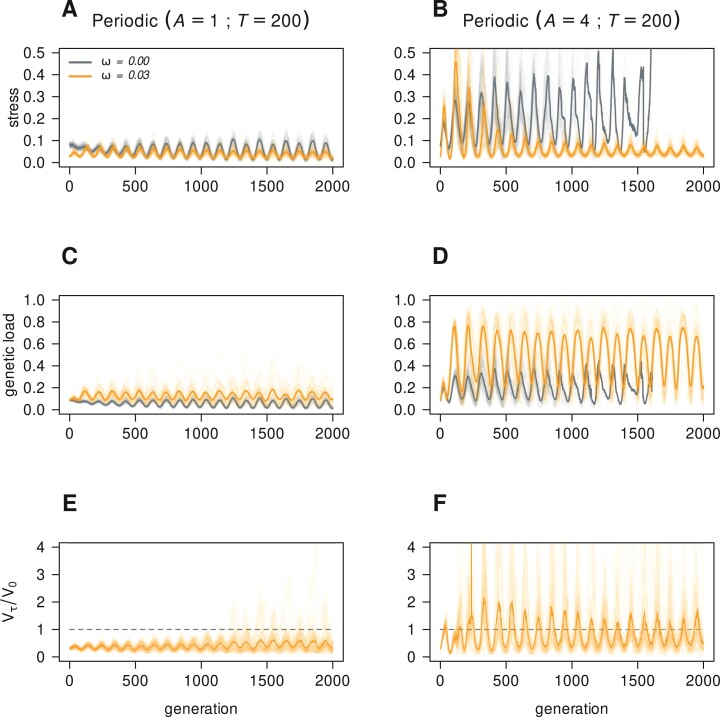
Evolution of 30 plastic (ω=0.03) and 30 non-plastic (ω=0) populations under moderate periodic (left column, amplitude and period on top) or extreme periodic (right column, amplitude and period on top) environmental change. The top row displays population stresses (Equation (8)), the middle row displays genetic loads (Equation (9)), and the bottom row shows phenotype variance ratios (Equation (10)). Ticker lines track averages. Parameters from Table 1.

**Figure 4:**
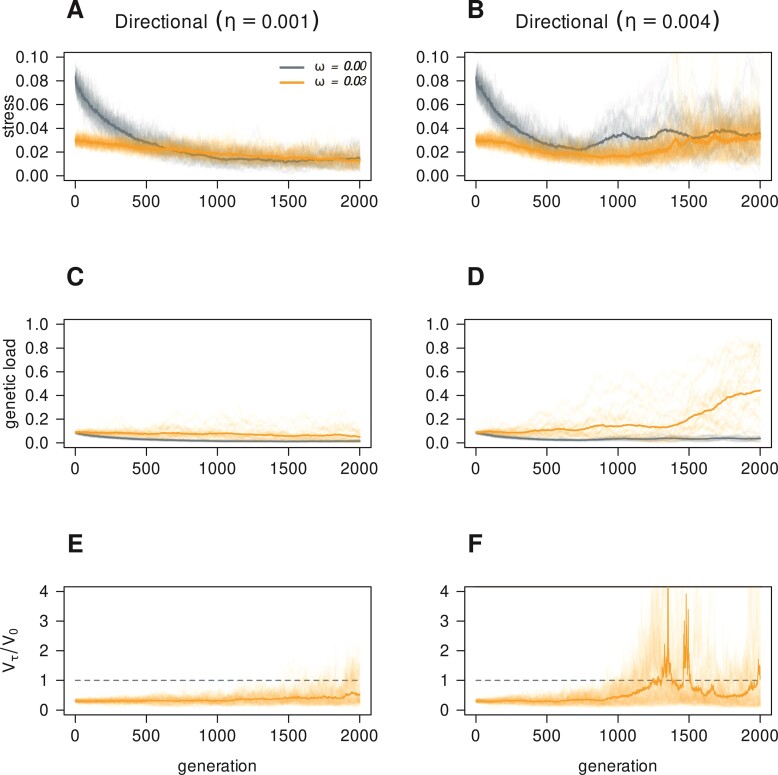
Evolution of 30 plastic (ω=0.03) and 30 non-plastic (ω=0) populations, under moderate directional (left column, rate on top) or extreme directional (right column, rate on top) environmental change. The top row displays population stresses (Equation (8)), the middle row displays genetic loads (Equation (9)), and the bottom row shows phenotype variance ratios (Equation (10)). Ticker lines track average tendencies. Parameters fromTable 1.

We wanted to assess the effect of plasticity on population dynamics and evolution. This was accomplished by simulating plastic (ω=0.03) and non-plastic (ω=0) populations during 2,000 generations. Genotypes are generated by recombination of parental genotypes from the previous generation with allelic mutation ($\mu_{m}=10^{-4}$). Evolution takes place under periodic (Equation (6), with period T=200) or directional (Equation (7)) regimes of environmental change. The amplitude of periodic change was set at A=1, and the rate of directional change at η=0.001. This means that the amount of directional change matches the amplitude of periodic change in the 1,000th generation (or the periodic min–max range in the 2,000th). To explore the limits of phenotypic plasticity by epigenetic mutations, we also considered more extreme environmental changes. We multiplied the amplitude of periodic environmental variation and the rate of directional change by 4 and repeated the simulations (right column of Figures 3 and 4, respectively). Each {plastic, non-plastic}×{periodic, directional} combination was replicated 30 times. Figures 3 and 4 show the outcome of these simulations.

Population performance was assessed every generation by averaging the stress metric (5) of all individuals


$$Stress=\frac{\sum_{i=1}^{N}\rho \left(x_{i}\right)}{N}.$$ (8)

We also quantified the genetic load of the populations


$$Genetic\,load=\frac{W_{max}^{o}-{\bar{W}}^{o}}{W_{max}^{o}},$$ (9)

where ${\bar{W}}^{o}$ and $W_{max}^{o}$ are, respectively, the average and the highest fitness in a population without epigenetic alterations (i.e., $a_{j}=1$ in Equation (1)). A large genetic load means that a population consists of relatively many sub-optimal genotypes and relatively few genotypes with high fitness value (regardless of $W_{max}^{o}$ being actually adequate or not in the current generation).

The efficiency of the plasticity mechanism was assessed in each generation by the ratio of phenotype variance between start (o) and end (τ) of development


$$\frac{V_{\tau }}{V_{0}}=\frac{\sum_{i=1}^{N}{\left(x_{i,\tau }-{\bar{x}}_{\tau }\right)}^{2}}{\sum_{i=1}^{N}{\left(x_{i,o}-{\bar{x}}_{o}\right)}^{2}},$$ (10)

$V_{\tau }/V_{0}<1$ indicates that epigenetics drives a large number of genotypes toward common trait values, that is, phenotypes are *canalized* to match the environmental parameter p in the current generation. Increase in $V_{\tau }/V_{0}$ ratios indicate that trait distributions are becoming a more wider or multimodal as a result of epigenetics. $V_{\tau }/V_{0}>1$ indicates that epigenetic mutations are far too frequent ($\mu_{e}$ too large), leading to extreme phenotype variability.

When the environment changes periodically plastic and non-plastic populations alternate between low and high stress (Figure 3, panels A and B). Plastic populations tend to display lower stress levels compared with non-plastic, and differences are wider when the environment fluctuations are extreme. Indeed, under extreme periodic fluctuations, non-plastic populations experience stress levels that could be large enough to drive them toward extinction (panel B). Plastic populations accumulate larger genetic loads compared with non-plastic (panel C). This indicates that epigenetic mutations prevent elimination of large numbers of alleles by natural selection. This is possible because the environment changes between finite lower and upper bounds, and there is enough genetic variability in the population for plasticity to work upon. Indeed, when changes in the environment are moderate, phenotype variance ratios of plastic populations remain constrained over many generations ($V_{t}/V_{0}<1$ on average, panel E), that is, populations are effectively canalized towards optimal phenotypes during periods of high and low stress. When changes are extreme though, genetic load in plastic populations attain very high levels (panel D), and the ability of epigenetic mutations to steer populations toward optimal phenotypes is lost after few generations (panel F).

Under directional change plastic and non-plastic populations maintain very low average stress levels (Figure 4, panels A and B). Speeding up environmental change (raising η) raises average stress levels for both population types. Genetic loads are low for both populations types but tend to be lower and decreasing for non-plastic (panels C and D). This indicates natural selection wiping out unfavorable alleles. Contrast this with plastic populations, where alleles can escape selection, allowing higher genetic loads (panel C), and its increase under rapid change (panel D). Like under periodic variation, plastic populations maintain low phenotype variance ratios, but there is an increasing trend nevertheless. This trend is weak under moderate environmental changes but becomes more evident when the rate of change is faster, leading to a sudden release of cryptic genetic variation, most of which is maladaptive (panel F), that is, trait adjusting by epigenetic mutations becomes less effective each generation. This likely happens because directional environmental change is asymmetric and unbounded: plastic populations with large genetic loads experience extreme environments with only a small fraction of individuals benefiting from epigenetic changes (those starting development close enough to optimum trait values), while the rest risk adaptation loss instead (due to developmental noise).

### Evolution of plasticity

**Figure 5:**
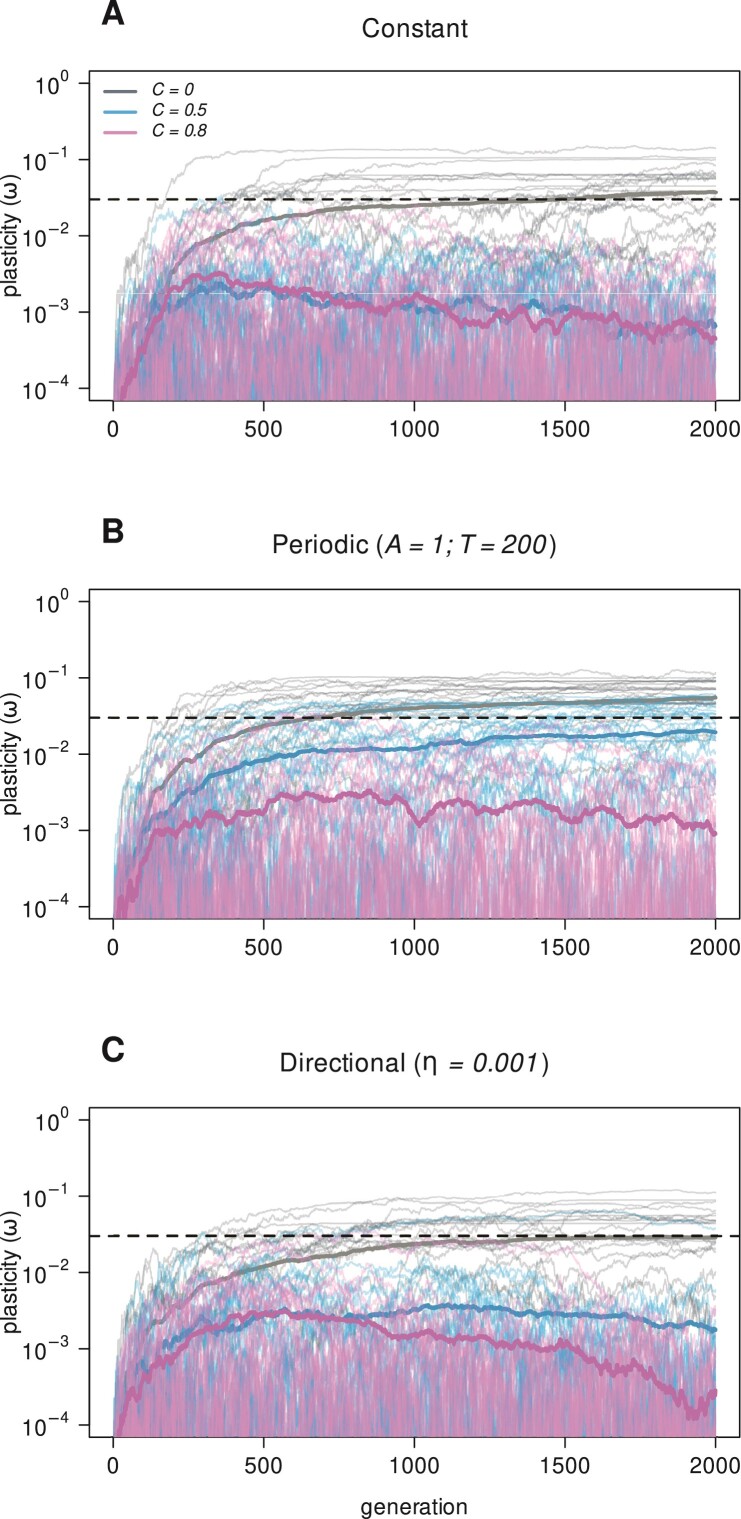
Evolutionary trajectories for sensitivity (plasticity) traits ω, with costs: none, moderate, or high (C = 0, 0.5, and 0.8, respectively). Thin lines are populations’ averages and thick lines display the main tendency (30 populations per cost value). The environmental parameter p is constant in A; periodic in B (A=1;T=200); or directionally changing in C (η=0.001). Parameters from Table 1.

We simulated population dynamics with genetically encoded ω (Equation (3)) in order to follow the evolution of plasticity itself. Without plasticity costs, sensitivity (plasticity) traits (ω) evolve and tend to stabilize above zero in the long term. This happens in constant environments, for (moderate) periodic changes, and for (moderate) directional change (shown in Figure 5 by the average trend, for C=0. Notice that the ω attained after 2,000 generations is of the same order of magnitude as in Figure 2B and C). Further evolution of ω halts because the benefits brought by epigenetic mutations, that is, developmental learning, are counteracted by the loss of function caused by developmental noise.

**Figure 6:**
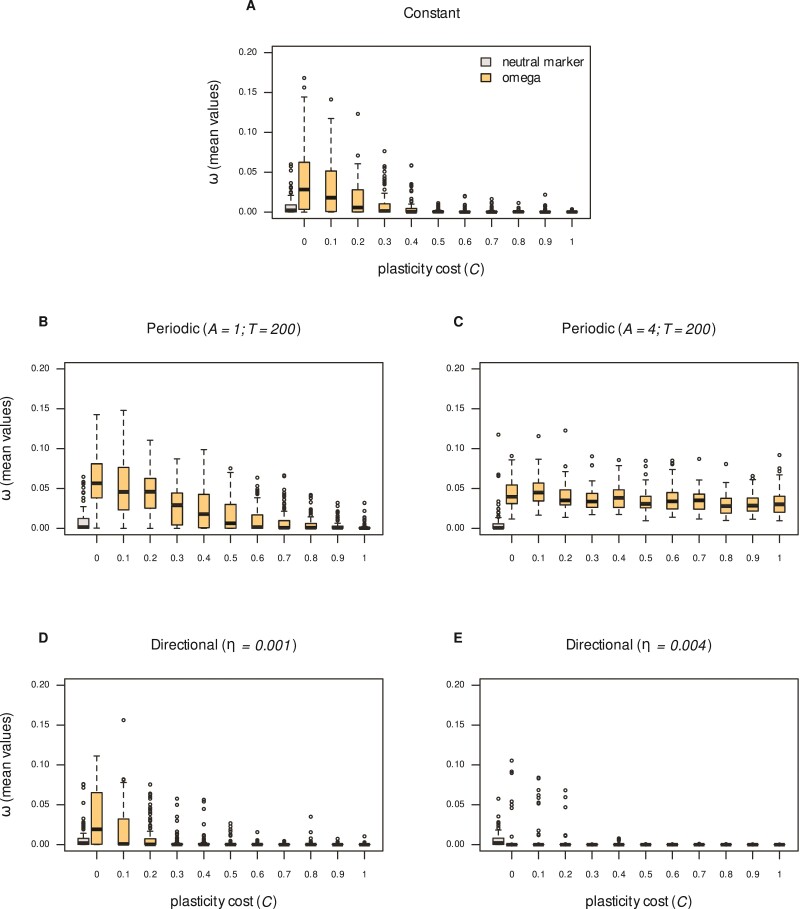
Sensitivity traits ω attained by evolution for different cost levels. Boxplots show distribution of ω averages for 100 populations after 2,000 generations. The distribution of a neutral marker (gray boxplot) serves as control. The environmental parameter p is constant in A; periodically changing (T=200) in B (A=1), C (A=4); or directional in D (η=0.001), E (η=0.004). Parameters from Table 1.

Plasticity costs are important in setting the extent of evolution: the higher the costs, the lower the long term ω (Figure 6, see also temporal trends in Figure 5). Costs have much higher impacts under directional environmental change in comparison with periodic change. If the environment changes at a moderate pace, ω values decline (near) uniformly with cost, but the drop is stronger under directional change compared with periodic (c.f., panels B and D). If directional changes are extreme, plasticity is not favored by selection, even at zero cost (panel E, compared with the neutral marker distribution). In stark contrast, under extreme periodic fluctuations sensitivity traits are favorably selected even at very high costs (panel C, ω declines eventually with C, but is not shown).

We also simulated the evolution of ω for asexual haploid populations (using simple versions of Equations (1 and 3) and without allele recombination). We found that the sensitivity (plasticity) trait is severely selected against in constant or directionally changing environments, and favored if the environment changes periodically, in a similar fashion like Figure 6 (see Supplementary Figure A.8).

## Discussion

This study demonstrates a novel mechanism in which epigenetic mutations enable phenotypic plasticity. This is achieved through random changes in the expression of multiple loci (or alternatively CpG sites in a locus), in a process that simulates the silencing/activation of genes caused by DNA-methylation (and de-methylation). The frequency of epigenetic mutations correlates with the mismatch between the individual’s phenotype and the external environment. That is, there is feedback between genetic expression and individual stress. As a result, a large set of heterogeneous genotypes can converge toward common phenotypes that better fit the environment. Epigenetic changes also depend on an individual’s genotype; thus, plasticity itself can be subject to natural selection.

Our simulations predict that epigenetic mutations improve the match between individuals and the environment. This is also the case for populations under periodic or directional regimes of environmental change. However, the effectiveness of epigenetic mutations decreases if environmental changes are too extreme. This is because epigenetic alterations also promote large genetic loads (i.e., preservation of less favorable alleles), combined with the fact that our proposed mechanism is inherently imperfect: random epigenetic mutations cannot distinguish good from bad loci activations. This has important consequences for the evolution of plasticity. We found that even in the absence of explicit costs associated with the epigenetic mechanism, the evolution of plastic genotypes remains bounded: the benefits of phenotype space exploration by trial and error (“learning-like behavior”) are balanced by the potential loss of function (developmental noise).

### Learning-like plasticity

In our model, individuals, that is, genotypes, adjust their phenotypes to match an environmental parameter p (e.g., osmotic pressure) by experiencing it before natural selection takes place, that is, during a finite development time in which the random motion governing the behavior of epigenetic markers (implied to be methyl groups that bind/unbind parts of the genome) produces adaptive plasticity. In reality, binds (deactivations) and unbinds (activations) are carried out by methyltransferases and demethylases, respectively, enzymes capable of adding or removing methyl radicals at specific places along DNA strands, affecting genetic expression (Moore et al., 2013; Shi et al., 2021). Thus, one could say that ω controls the synthesis of these enzymes, or the proportion that are active through allosteric regulation.

The adjustment of the phenotype is unpredictable, in the sense that individuals do not “know” which parts of the genotype, that is, which loci, must be activated and de-activated in order to increase fitness. Thus, adaptation simulates a process of “learning by trial and error” but with “memory loss.” A kind of “smart behavior” emerges due to a negative feedback on the rate of epigenesis (Equation (2)) during development: as the phenotype–environment mismatch |x−p| decreases, so does the frequency of epigenetic mutation $\mu_{e}$, and the chances of losing an adapted phenotype are minimized.

The fact that randomness enables the “learning-like” process described above suggests that phenotypic plasticity could evolve without the (hypothetically) complex and costly biochemistry required to activate and de-activate “only the right loci.” Thus, plasticity costs (Murren et al., 2015) can be very low, and random epigenetic mutations seem a plausible way to enable plasticity in simple, non-neural, unicellular organisms and in early life. It may also produce great morphological and physiological diversification in the face of highly conserved core genetic, cell biological, and developmental processes (Kirschner & Gerhart, 1998) (not tested in this study). Yet, this study shows that, in contrast to other models of plasticity (e.g. Lande, 2009, 2014; Reed et al., 2010; Scheiner & Holt, 2012), perfect plasticity under negligible costs and high environmental predictability does not evolve.

For simplicity, our model considers a single phenotypic trait x and a set of loci that affect this trait either purely genetically (loci 1 to B) or by a combination of genetic and epigenetic effects (the remaining loci). We have shown that the epigenetic mutation rate can evolve to a value where a purely random process of placing epigenetic marks can markedly shift the trait x toward the optimal value p. The question arises as to whether this result still holds in the (more realistic) situation that (i) epigenetic mutations affect other genes (e.g., genes with “housekeeping tasks”) for which plasticity is more harmful than beneficial and/or (ii) that different traits need to match different environmental targets. Regarding (i), the results of our study would, obviously, be much less convincing if the epigenetic mutation rate would indiscriminately affect the functioning of all genes, including those that should stably encode the same phenotype, irrespective of the state of the environment. However, there is ample evidence that epigenetic marks are not placed indiscriminately and that the placement of these marks is related to the nucleotide sequence of the DNA (e.g. Adrian-Kalchhauser et al., 2020; Richards, 2006; Richards et al., 2017). In view of this, one would expect that natural selection on the DNA sequence will make “housekeeping” genes less susceptible to the placement of epigenetic marks. Regarding (ii), one solution is based on the fact that there is a diversity of epigenetic marks (e.g., Adrian-Kalchhauser et al., 2020). Different marks may be related to different “dimensions” of plasticity, that is, selection on the DNA sequence could make the loci encoding for trait x susceptible for epigenetic mark X and unsusceptible for mark Y, while the opposite happens for the loci encoding for trait y. Alternatively, several stress factors (perhaps even a large number) could together create an overarching level of stress (which might be a weighted average of the deviation of trait x from its optimal value p, of trait y from its optimal value q, etc.), which then, in turn, would regulate the epigenetic mutation rate for all loci determining x,y, etc. At present, much of this is speculative but an interesting topic for future investigations.

### Genetic limits and emerging fitness costs to plasticity

Our study demonstrates two types of limits to phenotypic plasticity: (a) a *genetic limit* to plasticity, and (b) an *intrinsic limit* of the plasticity mechanism itself.

In nature, genomes are of finite size and this already imposes genetic limits to the range of phenotypes an individual organism can attain by means of its plasticity mechanism. Let us say that X is the set of possible phenotypes. Assuming $x_{opt}\in X$, the intrinsic limit consists of the likelihood that $x_{opt}$ will be attained and not lost during the development phase due to epigenetic mutations. This depends on the sensitivity ω (see Figure 2) and the trait–environment mismatch |x−p|. Note that this limitation does not emerge because the plasticity machinery is energetically costly, but rather because the mechanism itself is vulnerable to catastrophic error if ω is too high, or under extreme stress. In this case, developmental noise limits the ability of a plastic individual to match the optimal phenotype (DeWitt et al., 1998).

In this study, alleles constitute the raw material that the mechanism uses to develop phenotypic responses. According to our model, the genetic limits of plasticity can be expanded—though not necessarily always—by increasing the number of plastic loci L−B, for example by the insertion of transposable elements (Slotkin & Martienssen, 2007). In the model, each individual [genotype] has $2^{L-B}$ potential phenotypes thanks to epigenetic mutations (i.e., L−B binary states, active/inactive). Varying the number of plastic loci affected the degree of plasticity and effectiveness of the plasticity mechanism (see Supplementary Figures A.4, A.6, and A.7). Further study is needed to reveal why our model leads to a different conclusion than the study of Scheiner and Holt (2012) that found, that the number of non-plastic loci—but not the number of plastic ones—affects selection on plasticity.

Since the model uses real numbers for the allelic values, the condition all L loci active does not necessarily yields a maximum phenotypic value (e.g., maximum character size or length). However, it shows that the spectrum of phenotypic possibilities an individual can potentially attain during trait development, though diverse, is genetically limited by its genotype. It follows as well, that many loci of small effects can enable smoother phenotypic tracking of fluctuations in p, while few, stepwise, discrete responses.

Furthermore, it also demonstrates that different genotypes can have, to some extent, an *intersecting set* of phenotypic responses due to plasticity. The epigenetic mechanism can canalize such genetic variation into narrower phenotypic outcomes, which makes the optimal—wild type—phenotype mutationally robust (Hermisson & Wagner, 2004) (e.g., Figure 3C and E). The fact that genetically different individuals can still produce similar phenotypes enables the population to display coexistence of genetic polymorphisms, highly maintained genetic variation, and many-to-one genotype–phenotype maps (Wagner, 2005). Altogether, these features can enhance the evolvability of biological systems (Wagner, 2008). However, the coexistence of genetic polymorphisms due to the accumulation of mutations goes parallel with the accumulation of genetic load, and eventually with the loss of efficiency for the epigenetic mutation mechanism under strong regimes of environmental change (right column of Figures 3 and 4). Finally, the mechanism can make the same genotype susceptible to yield divergent phenotypic outcomes (e.g., when either, ω or trait–environment difference |x−p| is too large, Equation (2)).

The model shows that plastic populations, when not able to minimize their mismatch with the environment (i.e., large $\left|\bar{x}-p\right|$), release phenotypic variation, most of which is maladaptive. We observed this for directional trends, but may occur under periodic trends as well if the amplitude of fluctuations goes far beyond the population capability of producing suitable phenotypic adjustments (see Figures 3 and 4). Although the plasticity mechanism can effectively canalize populations toward optimal phenotypes, it inevitably leads as well toward increased genetic loads (see Figures 3 and 4). This variation is hidden, in the sense that it is already present in the population, and unleashed explosively (Figures 3F and 4F) due to large epigenetic mutation rates ($\mu_{e}$) failing to attain or keep suitable phenotypes. Under such extreme trends, plasticity hampers evolution. This provides an additional explanation to the often empirically observed cryptic genetic variation (Paaby & Rockman, 2014): a phenotypic outcome of the population (with high genetic load) that results from frequent failures of the plasticity mechanism in attaining the adaptive phenotype. Though to our model most of this variation is maladaptive, under other plasticity mechanisms (e.g., gene regulatory networks; van Gestel & Weissing, 2016), cryptic variation can be interpreted as opportunities potentially enhancing evolvability.

### Evolution of the plasticity mechanism

The evolutionary simulations show that the plasticity mechanism introduced in this study can evolve in populations, starting from the non-plastic condition (ω=0). The evolution of plasticity (*sensitivity trait*) is bounded by developmental noise and by the cost of the plasticity mechanism.

Our simulations indicate that periodically fluctuating environments turn out to be the most favorable for the evolution of plasticity. This outcome may explain, for example, why the brackish water population of the snail *T. fluviatilis* displays a higher degree of plasticity than the freshwater counterpart. The salinity of the lakes inhabited by the fresh water populations is more constant along the year, as compared to the higher amplitude fluctuations of the salinity in brackish environments.

On the other hand and in agreement with previous theoretical studies, plasticity evolved under directional environmental change (Nunney, 2016; Scheiner et al., 2020). However, in contrast to our findings, under periodic environmental fluctuations, its evolution under directional environmental change was strongly impacted by plasticity costs and extreme environmental conditions. Thus, under the proposed mechanism, the potential importance of phenotypic plasticity in enhancing survival and in “buying time” for populations subject to directional—climate—change is questionable and demands empirical verification.

We performed our simulations under the assumption of sexual individuals, where genotypes are re-shuffled each generation due to allele recombination. Yet, our evolutionary simulations for haploids (see Supplementary Figure A.8) lead to the same general conclusion that plasticity is considerably more favored by natural selection under regimes of periodic rather than directional environmental change (or not changing at all). This could make our predictions testable by employing haploid laboratory models, for example, bacterial strains.

### Conclusions

This study demonstrates that a classical explicit genetic model of quantitative genetics (Bürger & Lynch, 1995; Lynch et al., 1998; Scheiner et al., 2020) accounting for epigenetic mechanisms can produce adaptive, learning-like phenotypic plasticity; and, in-line with other mechanistic models (e.g., Brun-Usan et al., 2020a; van Gestel & Weissing, 2016; Wagner, 1994), developmental canalization of the genetic variation, many-to-one genotype–phenotype maps, mutational robustness of the wild-type phenotype, and the uncovering of cryptic variation. For previous mechanistic models, such attributes of the plasticity mechanism occur when assuming topological complexity in the form of number and types of regulatory connections (e.g., gene-regulatory networks; Brun-Usan et al., 2020b; van Gestel & Weissing, 2016). This study expands this view and shows that they can as well result from random epigenetic mutations occurring on a trivial genotype–phenotype mapping (i.e., loci with additive interactions only). In addition, our mechanism produces: still functional phenotypic responses under novel environmental conditions (not previously screened by selection), genetic limits of plasticity, and emerging fitness costs to plasticity. All these features appear in the model under relatively simple underlying assumptions (i.e., random motion governing the regulatory process). These simple rules enable improving adaptation by a learning-like process.

Our mechanism is motivated by the following conceptualization of phenotypic plasticity: plasticity consists of the phenotypic change by which an organism attempts to return to a referential state (e.g., osmotic balance). Conserving such a state prevents function loss and, ultimately, favors increased fitness for plastic organisms. This is analogous to *Le Chatelier’s principle* in chemistry, where “if a change is made to a system, the system will respond such as to absorb the force causing the change” (Anderson, 2005).

Given that the mechanism in this study requires developmental time and “trial and error,” this study speculates that deterministic and exploratory mechanisms might be evolutionarily linked: faster and reliable deterministic mechanisms can accommodate (into complex topology) solutions found by exploratory mechanisms, which are typically prone to errors and may require longer developmental time.

## Supplementary Material

qrae012_suppl_Supplementary_Figures_S1-S8

## Data Availability

Data and code available on github: https://github.com/danielrm84

## References

[CIT0001] Adrian-Kalchhauser, I., Sultan, S. E., Shama, L. N., Spence-Jones, H., Tiso, S., Valsecchi, C. I. K., & Weissing, F. J. (2020). Understanding “non-genetic” inheritance: Insights from molecular-evolutionary crosstalk. Trends in Ecology & Evolution, 35(12):1078–1089. 33036806 10.1016/j.tree.2020.08.011

[CIT0002] Anderson, G. M. (2005). Thermodynamics of natural systems. Cambridge University Press.

[CIT0003] Angers, B., Perez, M., Menicucci, T., & Leung, C. (2020). Sources of epigenetic variation and their applications in natural populations. Evolutionary Applications, 13(6):1262–1278. 32684958 10.1111/eva.12946 PMC7359850

[CIT0004] Ashander, J., Chevin, L.-M., & Baskett, M. L. (2016). Predicting evolutionary rescue via evolving plasticity in stochastic environments. Proceedings of the Royal Society B: Biological Sciences, 283(1839):20161690. 10.1098/rspb.2016.1690 PMC5046909 27655762

[CIT0005] Barton, N. H., Etheridge, A. M., & Véber, A. (2017). The infinitesimal model: Definition, derivation, and implications. Theoretical Population Biology, 118:50–73. 28709925 10.1016/j.tpb.2017.06.001

[CIT0006] Berrigan, D., & Scheiner, S. M. (2004). Modeling the evolution of phenotypic plasticity. Phenotypic plasticity: Functional and conceptual approaches (pp. 82–97). New York, NY, Oxford University Press.

[CIT0007] Botero, C. A., Weissing, F. J., Wright, J., & Rubenstein, D. R. (2015). Evolutionary tipping points in the capacity to adapt to environmental change. Proceedings of the National Academy of Sciences of the USA, 112(1):184–189. 25422451 10.1073/pnas.1408589111 PMC4291647

[CIT0008] Brun-Usan, M., Rago, A., Thies, C., Uller, T., & Watson, R. A. (2020 a). Developmental models reveal the role of phenotypic plasticity in explaining genetic evolvability. bioRxiv.

[CIT0009] Brun-Usan, M., Thies, C., & Watson, R. A. (2020b). How to fit in: The learning principles of cell differentiation. PLoS Computational Biology, 16(4):e1006811. 32282832 10.1371/journal.pcbi.1006811 PMC7179933

[CIT0010] Bürger, R., & Krall, C. (2004). Quantitative-genetic models and changing environments. In R. Ferrière, U. Dieckmann, & D. Couvet (Eds.), Evolutionary Conservation Biology (Vol. 4, pp. 171–187). Cambridge University Press.

[CIT0011] Bürger, R., & Lynch, M. (1995). Evolution and extinction in a changing environment: A quantitative-genetic analysis. Evolution, 49(1):151–163. 28593664 10.1111/j.1558-5646.1995.tb05967.x

[CIT0012] Casadesús, J. (2016). Bacterial DNA methylation and methylomes. In R. Z. J. Albert Jeltsch. (Ed.), DNA methyltransferases-role and function (pp. 35–61). Springer. 10.1007/978-3-319-43624-1_3 27826834

[CIT0013] Chevin, L.-M., Lande, R., & Mace, G. M. (2010). Adaptation, plasticity, and extinction in a changing environment: Towards a predictive theory. PLoS Biology, 8(4):e1000357. 20463950 10.1371/journal.pbio.1000357 PMC2864732

[CIT0014] Curradi, M., Izzo, A., Badaracco, G., & Landsberger, N. (2002). Molecular mechanisms of gene silencing mediated by DNA methylation. Molecular and Cellular Biology, 22(9):3157–3173. 11940673 10.1128/MCB.22.9.3157-3173.2002 PMC133775

[CIT0015] DeWitt, T. J., Sih, A., & Wilson, D. S. (1998). Costs and limits of phenotypic plasticity. Trends in Ecology & Evolution, 13(2):77–81. 21238209 10.1016/s0169-5347(97)01274-3

[CIT0016] Duncan, E. J., Cunningham, C. B., & Dearden, P. K. (2022). Phenotypic plasticity: What has DNA methylation got to do with it? Insects, 13(2):110. 35206684 10.3390/insects13020110 PMC8878681

[CIT0017] Fallet, M., Luquet, E., David, P., & Cosseau, C. (2020). Epigenetic inheritance and intergenerational effects in mollusks. Gene, 729:144166. 31678264 10.1016/j.gene.2019.144166

[CIT0018] Foster, P. L. (2007). Stress-induced mutagenesis in bacteria. Critical Reviews in Biochemistry and Molecular Biology, 42(5):373–397. 17917873 10.1080/10409230701648494 PMC2747772

[CIT0019] Greenberg, M. V. C., & Bourc’his, D. (2019). The diverse roles of DNA methylation in mammalian development and disease. Nature Reviews Molecular Cell Biology, 20(10):590–607. 31399642 10.1038/s41580-019-0159-6

[CIT0020] Hattman, S., Kenny, C., Berger, L., & Pratt, K. (1978). Comparative study of DNA methylation in three unicellular eucaryotes. Journal of Bacteriology, 135(3):1156–1157. 99431 10.1128/jb.135.3.1156-1157.1978 PMC222496

[CIT0021] Hermisson, J. & Wagner, G. P. (2004). The population genetic theory of hidden variation and genetic robustness. Genetics, 168(4):2271–2284. 15611191 10.1534/genetics.104.029173 PMC1448756

[CIT0022] Hildebrandt, J.-P., Bleckmann, H., & Homberg, U. (2021). Penzlin-Lehrbuch der Tierphysiologie. Springer.

[CIT0023] Houle, D. (1992). Comparing evolvability and variability of quantitative traits. Genetics, 130(1):195–204. 1732160 10.1093/genetics/130.1.195 PMC1204793

[CIT0024] Huey, R. B., Hertz, P. E., & Sinervo, B. (2003). Behavioral drive versus behavioral inertia in evolution: A null model approach. The American Naturalist, 161(3):357–366. 10.1086/346135 12699218

[CIT0025] Jablonka, E. (2017). The evolutionary implications of epigenetic inheritance. Interface Focus, 7(5):20160135. 28839916 10.1098/rsfs.2016.0135 PMC5566804

[CIT0026] Kirschner, M. & Gerhart, J. (1998). Evolvability. Proceedings of the National Academy of Sciences, 95(15):8420–8427. 10.1073/pnas.95.15.8420 PMC33871 9671692

[CIT0027] Kribelbauer, J. F., Lu, X.-J., Rohs, R., Mann, R. S., & Bussemaker, H. J. (2020). Toward a mechanistic understanding of DNA methylation readout by transcription factors. Journal of Molecular Biology, 432(6):1801–1815. 31689433 10.1016/j.jmb.2019.10.021 PMC6961349

[CIT0028] Laland, K. N., Uller, T., Feldman, M. W., Sterelny, K., Müller, G. B., Moczek, A., Jablonka, E., & Odling-Smee, J. (2015). The extended evolutionary synthesis: Its structure, assumptions and predictions. Proceedings of the Royal Society B: Biological Sciences, 282(1813):20151019. 10.1098/rspb.2015.1019 PMC4632619 26246559

[CIT0029] Lande, R. (2009). Adaptation to an extraordinary environment by evolution of phenotypic plasticity and genetic assimilation. Journal of Evolutionary Biology, 22(7):1435–1446. 19467134 10.1111/j.1420-9101.2009.01754.x

[CIT0030] Lande, R. (2014). Evolution of phenotypic plasticity and environmental tolerance of a labile quantitative character in a fluctuating environment. Journal of Evolutionary Biology, 27(5):866–875. 24724972 10.1111/jeb.12360

[CIT0031] Li, E. & Zhang, Y. (2014). DNA methylation in mammals. Cold Spring Harbor Perspectives in Biology, 6(5):a019133. 24789823 10.1101/cshperspect.a019133 PMC3996472

[CIT0032] Lynch, M., & Walsh, B. (1998). Genetics and analysis of quantitative traits. (Vol. 1). Sinauer Sunderland, MA.

[CIT0033] Massicotte, R. & Angers, B. (2012). General-purpose genotype or how epigenetics extend the flexibility of a genotype. Genetics Research International, 2012:1–7. 10.1155/2012/317175 PMC3335555 22567383

[CIT0034] Moore, L. D., Le, T., & Fan, G. (2013). DNA methylation and its basic function. Neuropsychopharmacology, 38(1):23–38. 22781841 10.1038/npp.2012.112 PMC3521964

[CIT0035] Murren, C. J., Auld, J. R., Callahan, H., Ghalambor, C. K., Handelsman, C. A., Heskel, M. A., Kingsolver, J., Maclean, H. J., Masel, J., Maughan, H., Pfennig, D. W., Relyea, R. A., Seiter, S., Snell-Rood, E., Steiner, U. K., & Schlichting, C. D. (2015). Constraints on the evolution of phenotypic plasticity: Limits and costs of phenotype and plasticity. Heredity, 115(4):293–301. 25690179 10.1038/hdy.2015.8 PMC4815460

[CIT0036] Niederhuth, C. E., Bewick, A. J., Ji, L., Alabady, M. S., Kim, K. D., Li, Q., Rohr, N. A., Rambani, A., Burke, J. M., Udall, J. A., Egesi, C., Schmutz, J., Grimwood, J., Jackson, S. A., Springer, N. M., & Schmitz, R. J. (2016). Widespread natural variation of DNA methylation within angiosperms. Genome Biology, 17(1):1–19. 27671052 10.1186/s13059-016-1059-0 PMC5037628

[CIT0037] Nunney, L. (2016). Adapting to a changing environment: Modeling the interaction of directional selection and plasticity. Journal of Heredity, 107(1):15–24. 26563131 10.1093/jhered/esv084

[CIT0038] Paaby, A. B., & Rockman, M. V. (2014). Cryptic genetic variation: Evolution’s hidden substrate. Nature Reviews Genetics, 15(4):247–258. 10.1038/nrg3688 PMC4737706 24614309

[CIT0039] Parsons, K. J., McWhinnie, K., Pilakouta, N., & Walker, L. (2020). Does phenotypic plasticity initiate developmental bias? Evolution & Development, 22(1–2):56–70. 31348849 10.1111/ede.12304 PMC7004013

[CIT0040] Pfennig, D. W. (2021). Phenotypic plasticity & evolution: Causes, consequences, controversies. Taylor & Francis.

[CIT0041] Pigliucci, M. (2005). Evolution of phenotypic plasticity: Where are we going now? Trends in Ecology & Evolution, 20(9):481–486. 16701424 10.1016/j.tree.2005.06.001

[CIT0042] Reed, T. E., Waples, R. S., Schindler, D. E., Hard, J. J., & Kinnison, M. T. (2010). Phenotypic plasticity and population viability: The importance of environmental predictability. Proceedings of the Royal Society B: Biological Sciences, 277(1699):3391–3400. 10.1098/rspb.2010.0771 PMC2982227 20554553

[CIT0043] Richards, C. L., Alonso, C., Becker, C., Bossdorf, O., Bucher, E., Colomé-Tatché, M., Durka, W., Engelhardt, J., Gaspar, B., Gogol-Döring, A., Grosse, I., van Gurp, T. P., Heer, K., Kronholm, I., Lampei, C., Latzel, V., Mirouze, M., Opgenoorth, L., Paun, O., Prohaska, S. J., Rensing, S. A., Stadler, P. F., Trucchi, E., Ullrich, K., & Verhoeven, K. J. F. (2017). Ecological plant epigenetics: Evidence from model and non-model species, and the way forward. Ecology Letters, 20(12):1576–1590. 29027325 10.1111/ele.12858

[CIT0044] Richards, E. J. (2006). Inherited epigenetic variation—revisiting soft inheritance. Nature Reviews Genetics, 7(5):395–401. 10.1038/nrg1834 16534512

[CIT0045] Romero-Mujalli, D., Rochow, M., Kahl, S., Paraskevopoulou, S., Folkertsma, R., Jeltsch, F., & Tiedemann, R. (2021). Adaptive and nonadaptive plasticity in changing environments: Implications for sexual species with different life history strategies. Ecology and Evolution, 11(11):6341–6357. 34141222 10.1002/ece3.7485 PMC8207414

[CIT0046] Scheiner, S. M., Barfield, M., & Holt, R. D. (2020). The genetics of phenotypic plasticity. XVII. Response to climate change. Evolutionary Applications, 13(2):388–399. 31993084 10.1111/eva.12876 PMC6976953

[CIT0047] Scheiner, S. M. & Holt, R. D. (2012). The genetics of phenotypic plasticity. X. Variation versus uncertainty. Ecology and Evolution, 2(4):751–767. 22837824 10.1002/ece3.217 PMC3399198

[CIT0048] Schlichting, C. D., & Wund, M. A. (2014). Phenotypic plasticity and epigenetic marking: An assessment of evidence for genetic accommodation. Evolution, 68(3):656–672. 24410266 10.1111/evo.12348

[CIT0049] Shi, J., Xu, J., Chen, Y. E., Li, J. S., Cui, Y., Shen, L., Li, J. J., & Li, W. (2021). The concurrence of DNA methylation and demethylation is associated with transcription regulation. Nature Communications, 12(1):5285. 10.1038/s41467-021-25521-7 PMC8421433 34489442

[CIT0050] Slotkin, R. K., & Martienssen, R. (2007). Transposable elements and the epigenetic regulation of the genome. Nature Reviews Genetics, 8(4):272–285. 10.1038/nrg2072 17363976

[CIT0051] Smithson, M., Thorson, J. L., Sadler-Riggleman, I., Beck, D., Skinner, M. K., & Dybdahl, M. (2020). Between-generation phenotypic and epigenetic stability in a clonal snail. Genome Biology and Evolution, 12(9):1604–1615. 32877512 10.1093/gbe/evaa181 PMC7513791

[CIT0052] Sommer, R. J. (2020). Phenotypic plasticity: From theory and genetics to current and future challenges. Genetics, 215(1):1–13. 32371438 10.1534/genetics.120.303163 PMC7198268

[CIT0053] Symanowski, F., & Hildebrandt, J.-P. (2010). Differences in osmotolerance in freshwater and brackish water populations of *Theodoxus fluviatilis* (gastropoda: Neritidae) are associated with differential protein expression. Journal of Comparative Physiology B, 180(3):337–346. 10.1007/s00360-009-0435-4 20012055

[CIT0054] Thibert-Plante, X., & Hendry, A. (2011). The consequences of phenotypic plasticity for ecological speciation. Journal of Evolutionary Biology, 24(2):326–342. 21091567 10.1111/j.1420-9101.2010.02169.x

[CIT0055] Thorson, J. L., Smithson, M., Beck, D., Sadler-Riggleman, I., Nilsson, E., Dybdahl, M., & Skinner, M. K. (2017). Epigenetics and adaptive phenotypic variation between habitats in an asexual snail. Scientific Reports, 7(1):1–11. 29074962 10.1038/s41598-017-14673-6 PMC5658341

[CIT0056] Thorson, J. L., Smithson, M., Sadler-Riggleman, I., Beck, D., Dybdahl, M., & Skinner, M. K. (2019). Regional epigenetic variation in asexual snail populations among urban and rural lakes. Environmental Epigenetics, 5(4):dvz020. 31723440 10.1093/eep/dvz020 PMC6836316

[CIT0057] van Gestel, J., & Weissing, F. J. (2016). Regulatory mechanisms link phenotypic plasticity to evolvability. Scientific Reports, 6(1):1–15. 27087393 10.1038/srep24524 PMC4834480

[CIT0058] Wagner, A. (1994). Evolution of gene networks by gene duplications: A mathematical model and its implications on genome organization. Proceedings of the National Academy of Sciences, 91(10):4387–4391. 10.1073/pnas.91.10.4387 PMC43790 8183919

[CIT0059] Wagner, A. (2005). Robustness and evolvability in living systems. Princeton University Press.

[CIT0060] Wagner, A. (2008). Robustness and evolvability: A paradox resolved. Proceedings of the Royal Society B: Biological Sciences, 275(1630):91–100. 10.1098/rspb.2007.1137 PMC2562401 17971325

[CIT0061] Watson, R. A. & Szathmáry, E. (2016). How can evolution learn? Trends in Ecology & Evolution, 31(2):147–157. 26705684 10.1016/j.tree.2015.11.009

