# Supplementary material for: Emergence of phenotypic plasticity through epigenetic mechanisms
Source: Evol Lett. 2024 Mar 27;8(4):561–74. doi: 10.1093/evlett/qrae012 (PMC11291936; doi:10.1093/evlett/qrae012)
Supplement: qrae012_suppl_Supplementary_Figures_S1-S8 [file qrae012_suppl_supplementary_figures_s1-s8.pdf]

Supplementary figures
Emergence of phenotypic plasticity through epigenetic mechanisms

Daniel Romero-Mujalli*[1,2,6], Laura I. R. Fuchs[1], Martin Haase[1], Jan-Peter Hildebrandt[1], Franz J. Weissing[3], and Tomás A. Revilla[4,5]

[1]Zoological Institute and Museum, University of Greifswald, Greifswald, Germany
[2]Institute for Botany and Landscape Ecology, University of Greifswald, Greifswald, Germany
[3]Groningen Institute for Evolutionary Life Sciences, University of Groningen, Groningen, The Netherlands
[4]Department of Mathematics, Faculty of Science, University of South Bohemia, České Budějovice, Czech Republic
[5]Czech Academy of Sciences, Biology Centre, Institute of Entomology, České Budějovice, Czech Republic
[6]Department for Environment Constructions and Design, Institute of Microbiology (IM), University of Applied Sciences and Arts of Southern Switzerland (SUPSI), Mendrisio, Switzerland (current address)

March 2, 2024

Abstract

Plasticity is found in all domains of life and is particularly relevant when populations experience variable environmental conditions. Traditionally, evolutionary models of plasticity are non-mechanistic: they typically view reactions norms as the target of selection, without considering the underlying genetics explicitly. Consequently, there have been difficulties in understanding the emergence of plasticity, and in explaining its limits and costs. In this paper, we offer a novel mechanistic approximation for the emergence and evolution of plasticity. We simulate random "epigenetic mutations" in the genotype–phenotype mapping, of the kind enabled by DNA-methylations/demethylations. The frequency of epigenetic mutations at loci affecting the phenotype is sensitive to organism stress (trait–environment mismatch), but is also genetically determined and evolvable. Thus, the "random motion" of epigenetic markers enables developmental learning-like behaviors that can improve adaptation within the limits imposed by the genotypes. However, with random motion being "goal-less", this mechanism is also vulnerable to developmental noise leading to maladaptation. Our individual-based simulations show that epigenetic mutations can hide alleles that are temporarily unfavorable, thus enabling cryptic genetic variation. These alleles can be advantageous at later times, under regimes of environmental change, in spite of the accumulation of genetic loads. Simulations also demonstrate that plasticity is favored by natural selection in constant environments, but more under periodic environmental change. Plasticity also evolves under directional environmental change as long as the pace of change is not too fast and costs are low.

*corresponding author, email: danielrm84@gmail.com

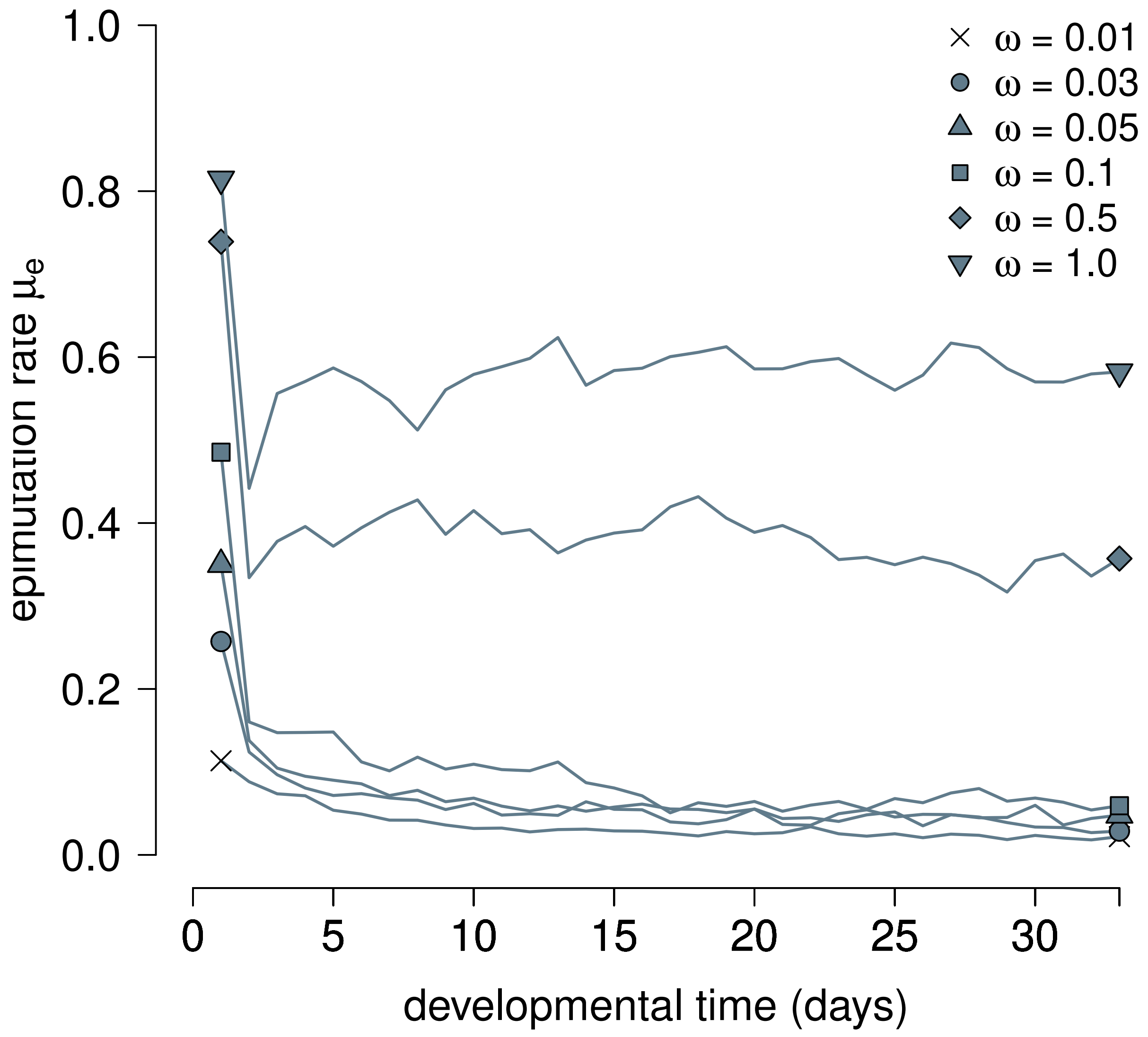

Figure A.1. Average change in the epimutation rate μ_e per scenario of sensitivity trait ω during the development of the response trait x in $N = 100$ populations.

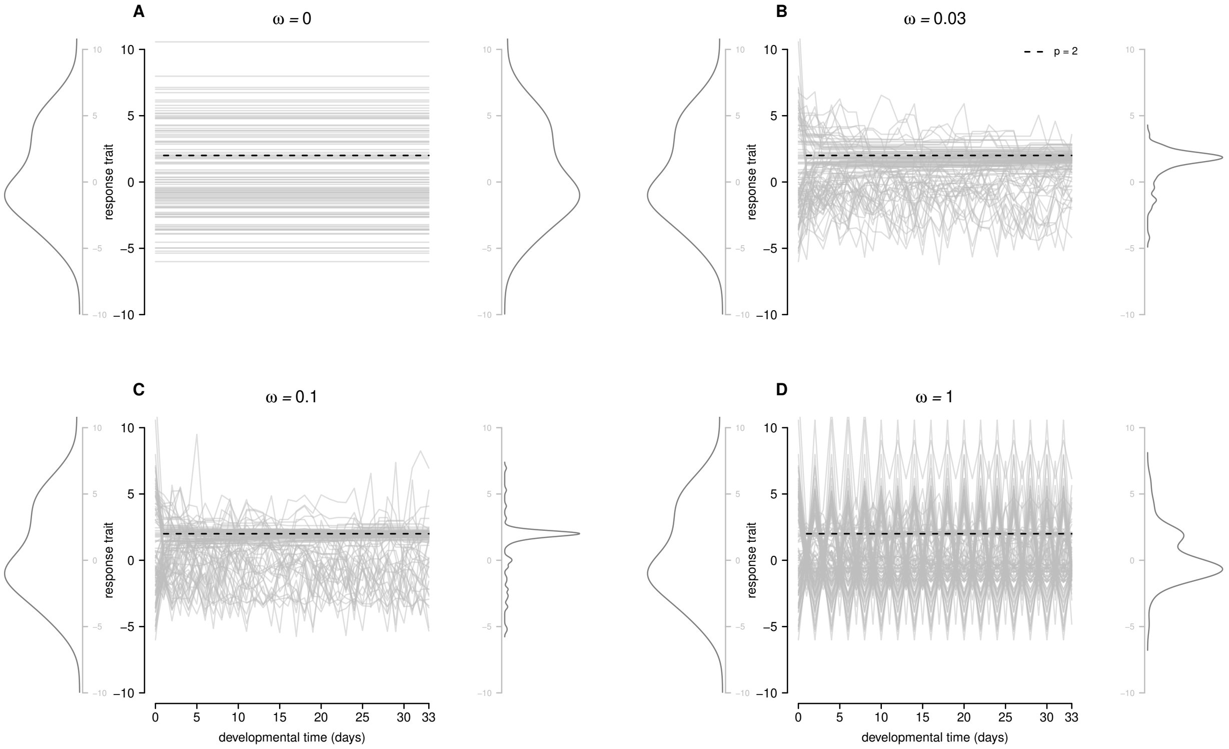

Figure A.2. Development of response traits x (eq. 1) for 100 individuals (the same in all panels), starting from an asymmetric trait distribution. The environmental parameter is $p = 2$, and the sensitivity trait is $\omega = 0, 0.03, 0.1, 1$, from panels A to D. Trait distributions at the start and end of development are displayed on the left and right side of each panel, respectively. Parameters from Table 1.

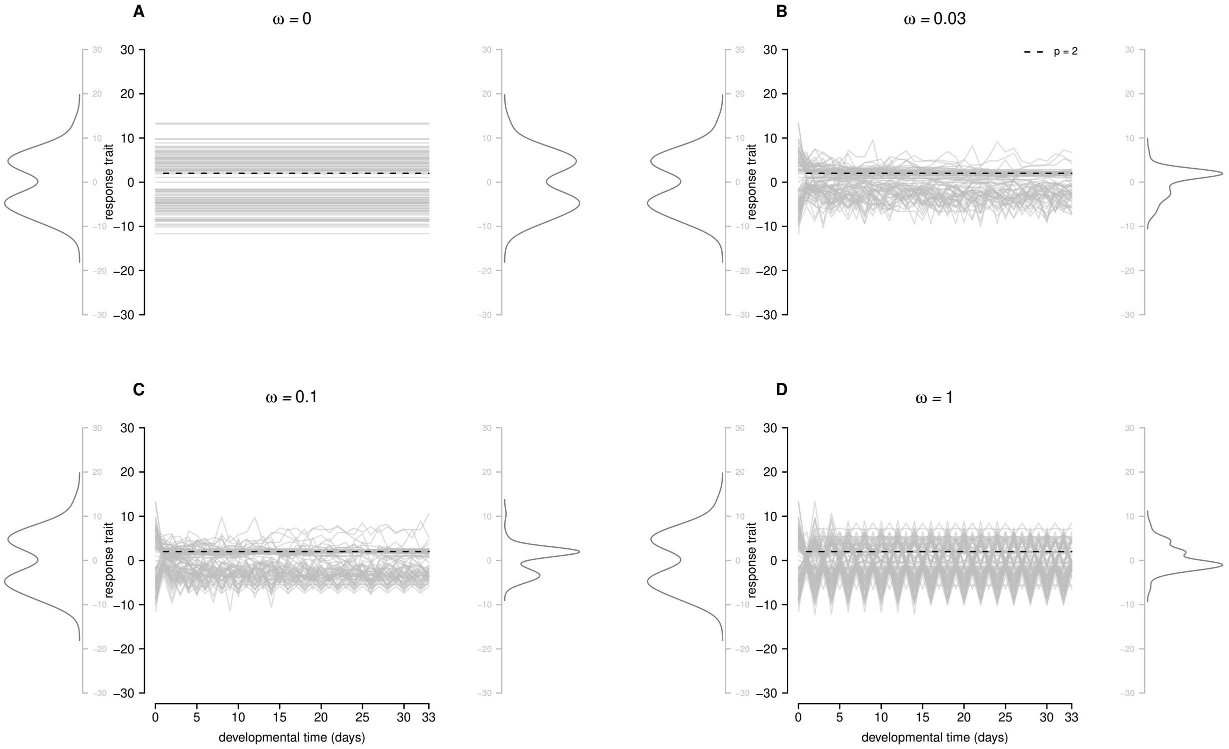

Figure A.3. Development of response traits x (eq. 1) for 100 individuals (the same in all panels), starting from a bimodal distribution. The environmental parameter is $p = 2$, and the sensitivity trait is $\omega = 0, 0.03, 0.1, 1$, from panels A to D. Trait distributions at the start and end of development are displayed on the left and right side of each panel, respectively. Parameters from Table 1.

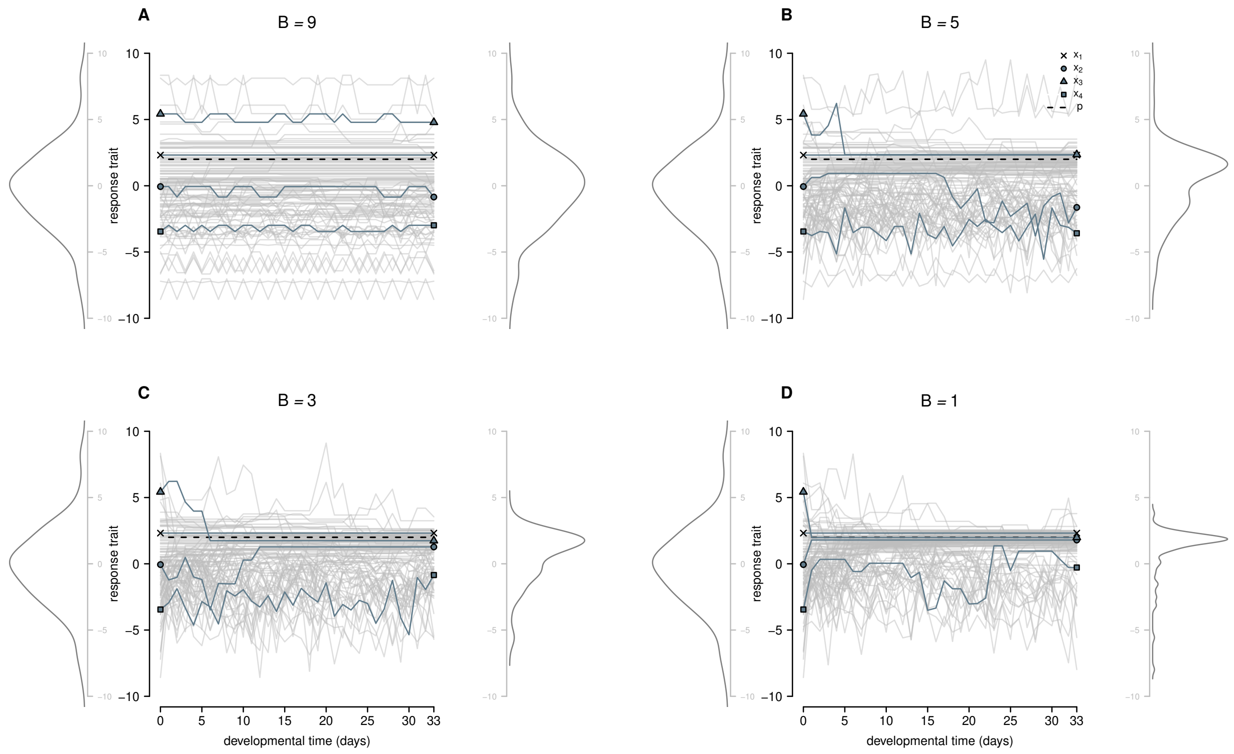

Figure A.4. Development of response traits x (eq. 1) for 100 individuals. The number of non-plastic loci B is indicated at the top of each panel (the total number of loci is $L = 10$). The sensitivity trait for epigenetic mutations is $\omega = 0.03$, and the environmental parameter $p = 2$. The development of four individuals ("cross", "circle", "triangle" and "square") is highlighted. Trait distributions at the start and end of the development are indicated on the left and right side of each panel, respectively. Parameters from Table 1.

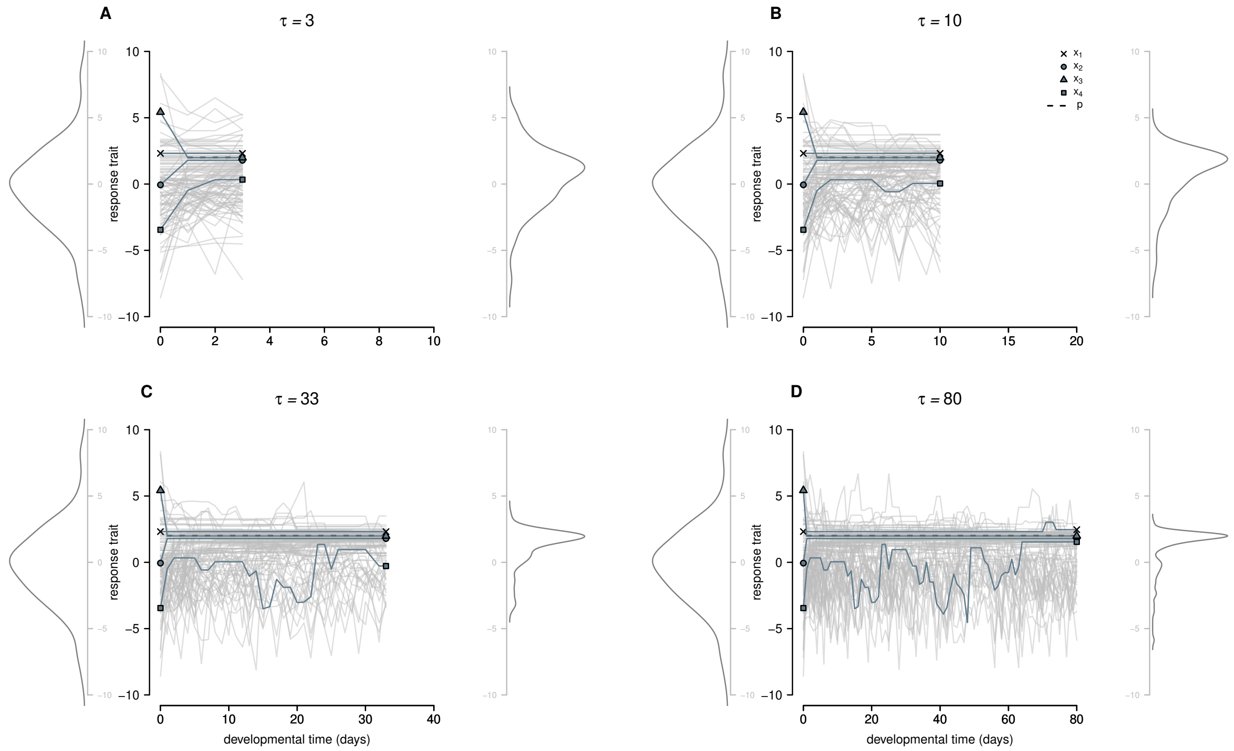

Figure A.5. Development of response traits x (eq. 1) for 100 individuals. The developmental time (τ) is indicated at the top of each panel. The sensitivity trait for epigenetic mutations is $\omega = 0.03$, and the environmental parameter $p = 2$. The development of four individuals ("cross", "circle", "triangle" and "square") is highlighted. Trait distributions at the start and end of the development are indicated on the left and right side of each panel, respectively. Parameters from Table 1.

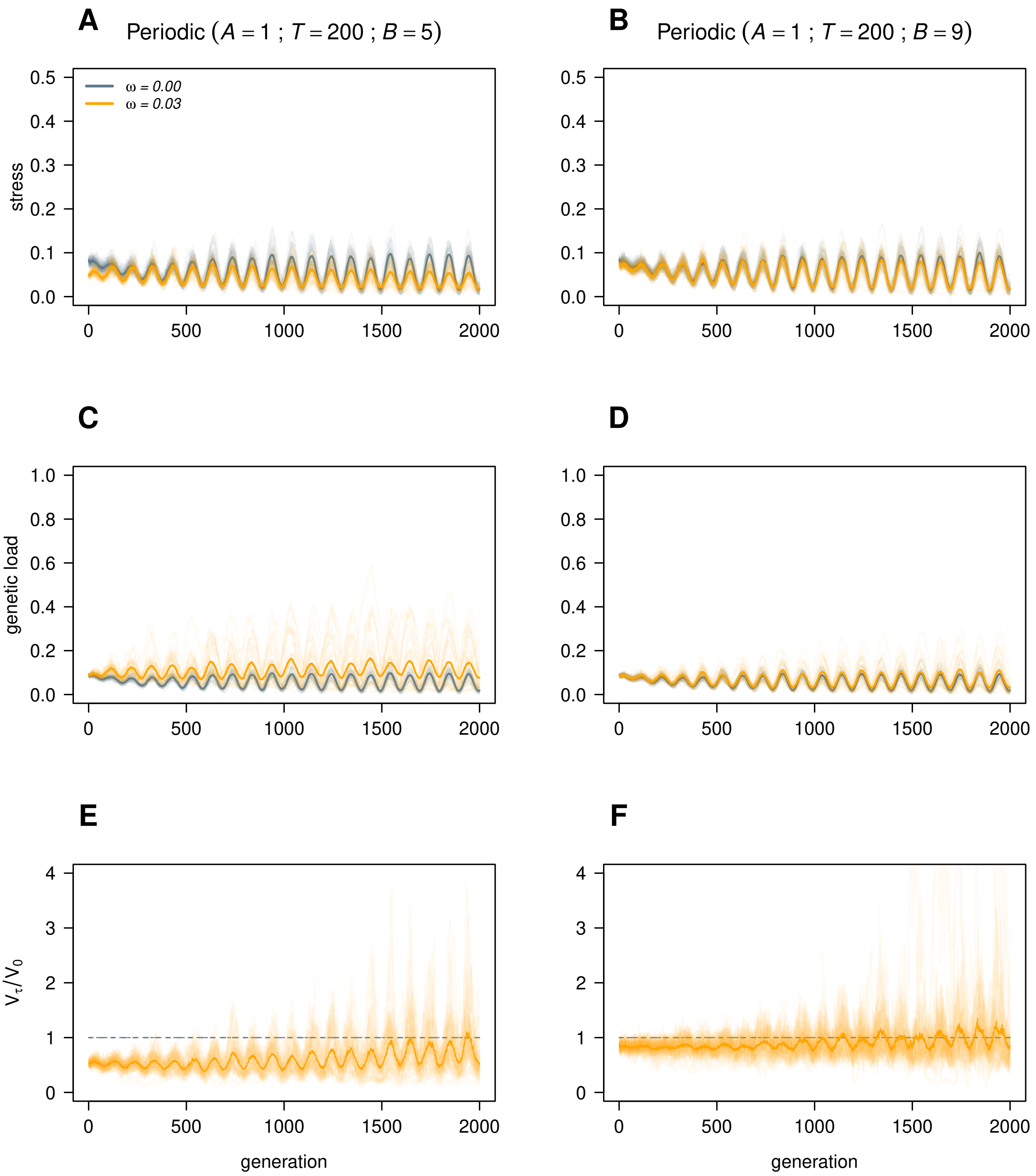

Figure A.6. Evolution 30 plastic (ω = 0.03, orange) and 30 non plastic (ω = 0, gray) populations with allelic mutations ($\mu_m = 10^{-4}$, panels C,D), under periodic ($A = 1; T = 200$) environmental change. 50%, left column, or 10%, right column, of the $L = 10$ trait loci were plastic (*i.e.*, B was either, 5 or 9). Parameters from Table 1.

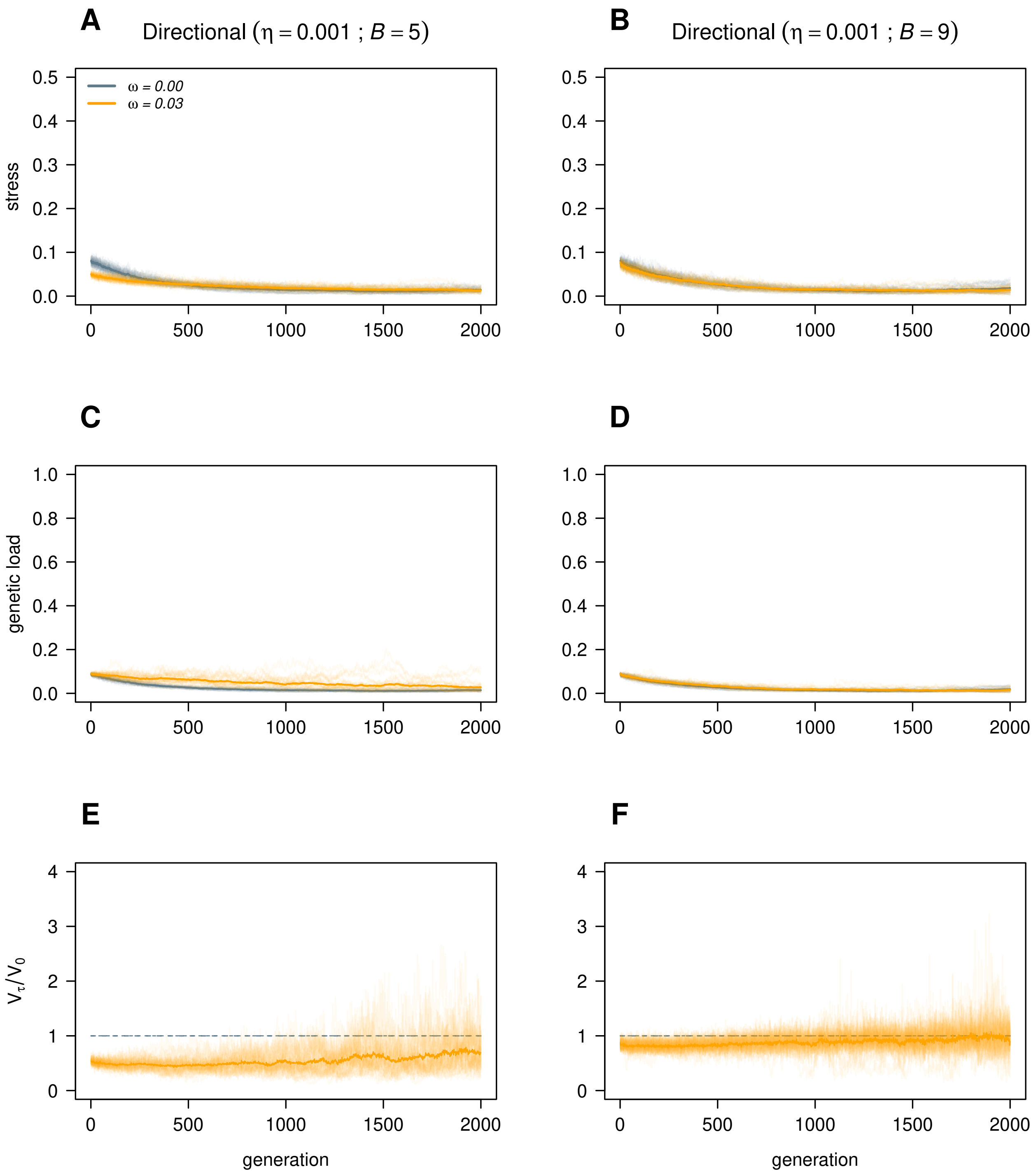

Figure A.7. Evolution 30 plastic ($\omega = 0.03$, orange) and 30 non plastic ($\omega = 0$, gray) populations with allelic mutations ($\mu_m = 10^{-4}$), under directional ($\eta = 0.001$) environmental change. 50%, left column, or 10%, right column, of the $L = 10$ trait loci were plastic (*i.e.*, B was either, 5 or 9). Parameters from Table 1.

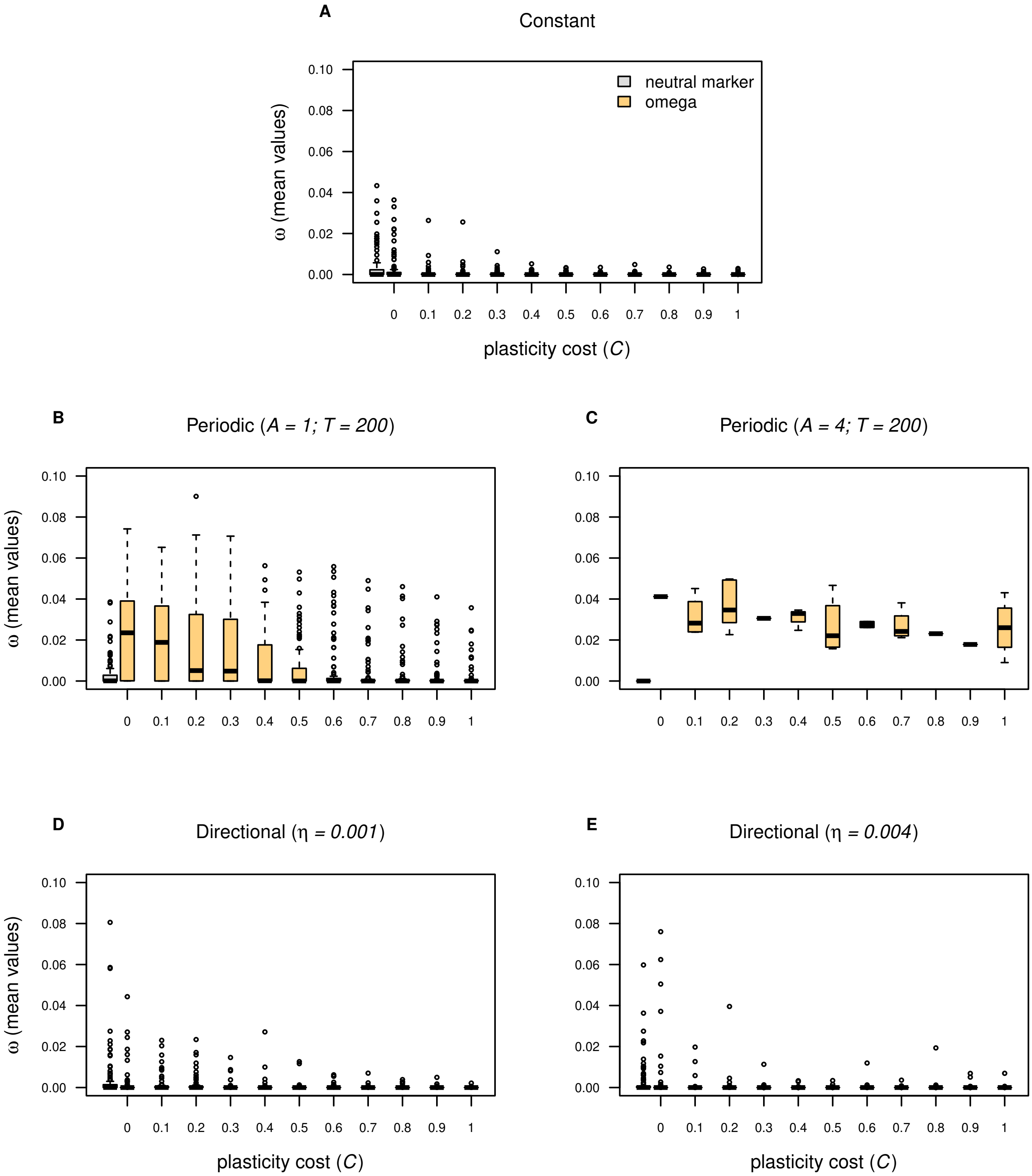

Figure A.8. Sensitivity traits ω attained by haploid populations vs. plasticity costs. Boxplots show distribution of average ω's for 100 populations after 2000 generations. The distribution of a neutral marker (gray boxplot) serves as control. The environmental parameter p is constant in A; periodic ($T = 200$) in B ($A = 1$), C ($A = 4$); or directionally changing in D ($\eta = 0.001$), E ($\eta = 0.004$). Parameters from Table 1.
